# Supplementary material for: Using pretrained graph neural networks with token mixers as geometric featurizers for conformational dynamics
Source: ArXiv. 2024 Dec 31:arXiv:2409.19838v2. Preprint. [Version 2] (PMC11722521)
Supplement: Supplement 1 [file NIHPP2409.19838v2-supplement-1.pdf]

TABLE S1. Hyperparameters for pretrained networks

| Hyperparameter | TorchMD-ET and ViSNet |
|---|---|
| $d$ | 64, 128, 256, 384 |
| # of MP layers | 6 |
| # of attention heads | 8 |
| Batch size | 100 |
| Epochs | 10 |
| # of RBFs | 64 |
| $r_{\text{cut}}$ (Å) | 5, 7.5 |
| Learning rate | 0.0005 |
| Optimizer | AdamW (AMSGrad) |
| Noise level (Å) | 0.2 |

TABLE S2. Hyperparameters for SPIB

| Hyperparameter | Shared | |
|---|---|---|
| Batch size | 1000 | |
| Learning rate | 0.0002 | |
| Optimizer | AdamAtan2 | |
| Training patience | 5 | |
| MLP hidden dimension | 64 | |
| MLP activation function | SiLU | |
| Embedding dimension ($d$) | 64 | |
| Dropout | 0.2 | |
| Batch normalization | False | |
| Label refinement frequency | 5 | |
| Architectures | SubFormer/SubMixer | |
| GVP | Yes | |
| # of GVP layers | 3 | |
| # of transformer/mixer layers | 3 | |
| Expansion factor | 2 | |
| Global token | True | |
| | **Villin** | **Trp-cage** |
| Trajectory stride | 2 | 4 |
| Training lag time (ns) | 20 | 10 |

TABLE S3. Hyperparameters for VAMPnets. For GVP variants tested on villin, the architecture is consistent with the ones used in SPIB tasks.

| Hyperparameter | Chignolin | Villin | Trp-cage |
|---|---|---|---|
| Batch size | 5000 | 5000/1000 | 5000/1000 |
| Learning rate | 0.0002 | 0.0002 | 0.0002 |
| Optimizer | AdamAtan2[81] | AdamAtan2 | AdamAtan2 |
| Maximum epochs | 20 | 20 | 20 |
| Training patience | 5 | 500 | 500 |
| Validation patience | 2 | 10 | 10 |
| Validation interval | 5 | 50 | 50 |
| MLP hidden dimension | 256 | 128/64 | 128/64 |
| MLP activation function | SiLU | SiLU | SiLU |
| Trajectory stride | 1 | 2 | 4 |
| Training lag time (ns) | 4 | 20 | 10 |
| Embedding dimension ($d$) | 256 | 128/64 | 128/64 |
| Dropout | 0.2 | 0.2 | 0.2 |
| Batch normalization | False | False | False |
| Output dimension ($d_o$) | 2 | 3 | 4 |
| Architectures | MLP/SubFormer/SubMixer | MLP/SubFormer/SubMixer | MLP/SubFormer/SubMixer |
| GVP | No | Yes | No |
| Expansion factor | 2 | 2 | 2 |
| Global token | False | True | False |

**Appendix E: Supplementary Figures**

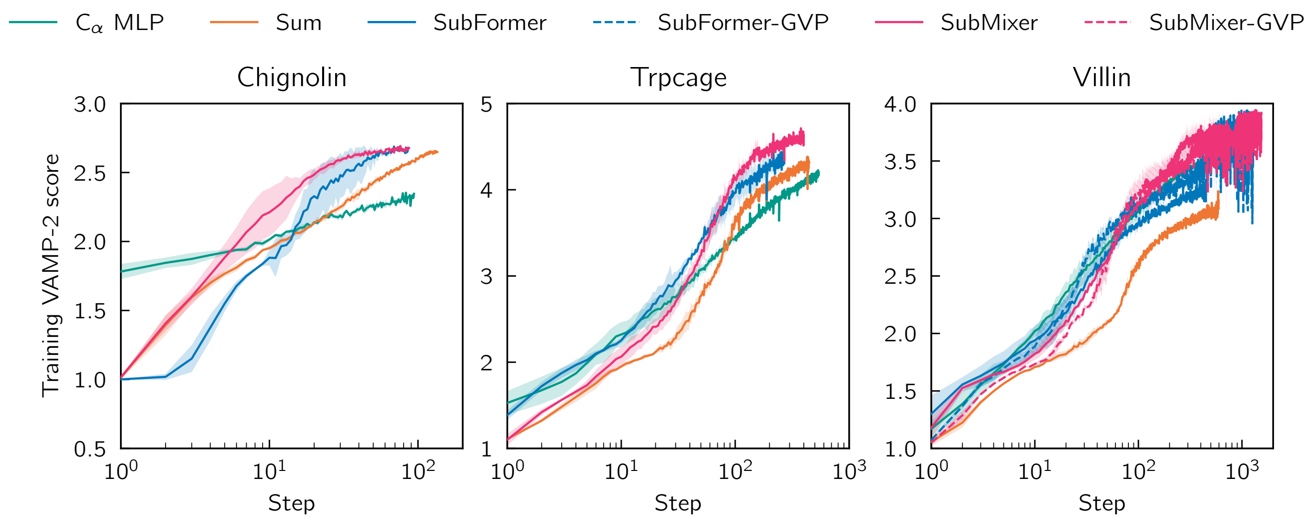


FIG. S1. Training curves for VAMPnets. Each curve represents the mean of three training runs. Shaded regions indicate standard error over three runs.

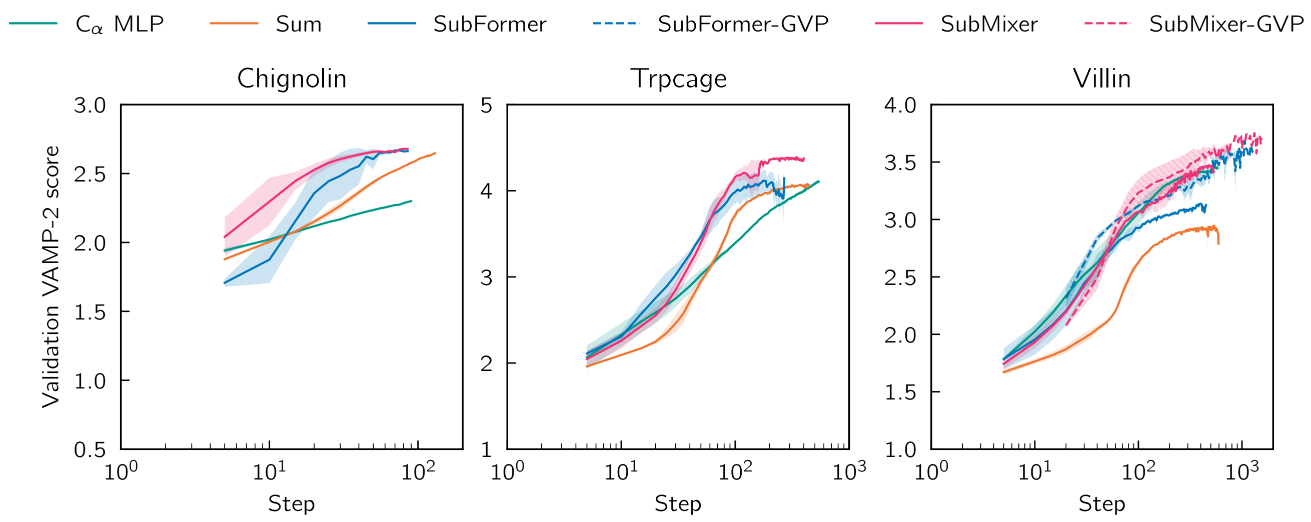


FIG. S2. Validation curves for VAMPnets. Each curve represents the mean of three training runs. Shaded regions indicate standard error over three runs.

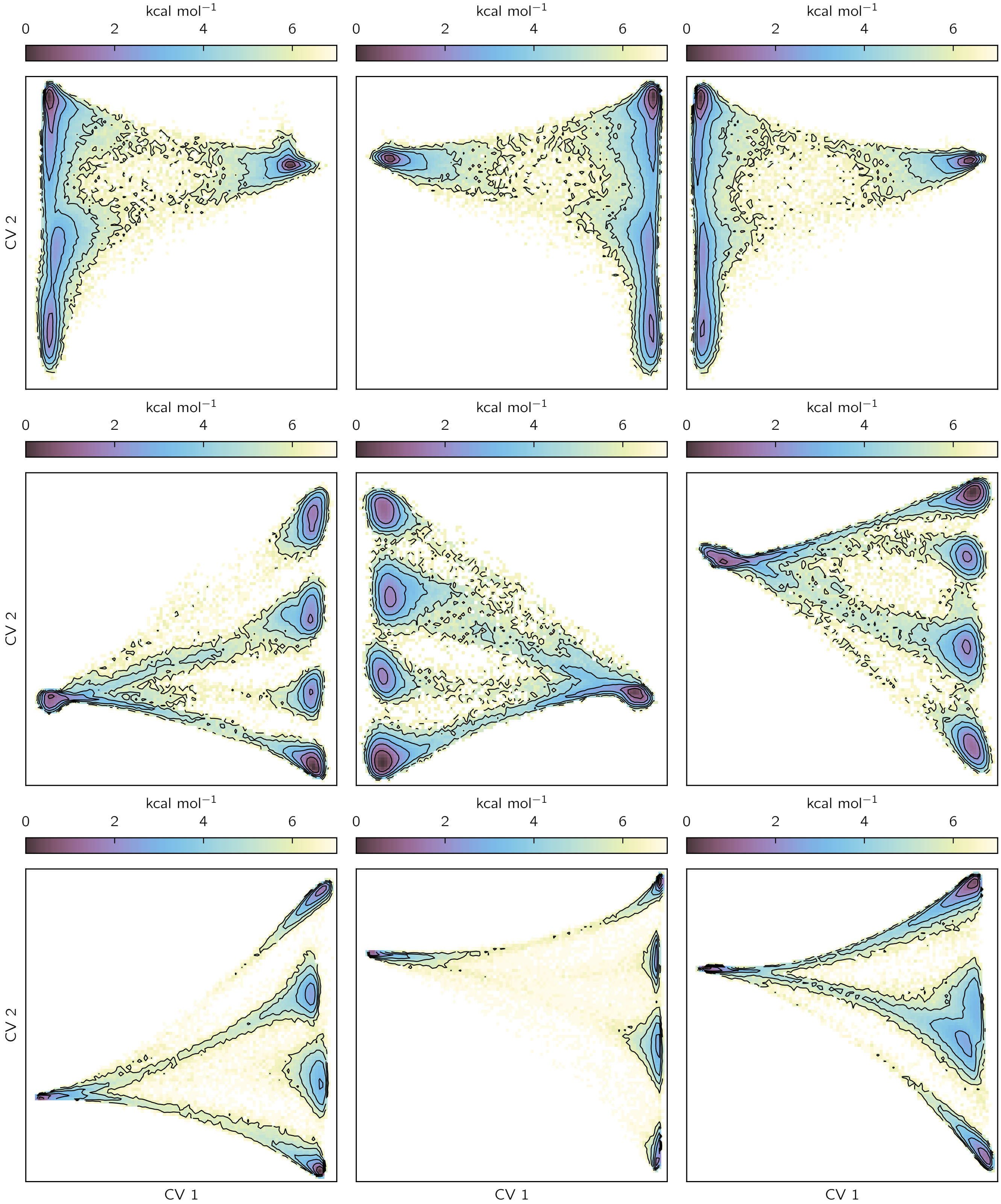


FIG. S3. PMFs as a function of VAMP CVs for chignolin. From top to bottom, VAMPnets were trained with no token mixer (Sum), SubMixer, or SubFormer. Each column shows the result from a single training run. Contours are drawn every 1 kcal/mol.

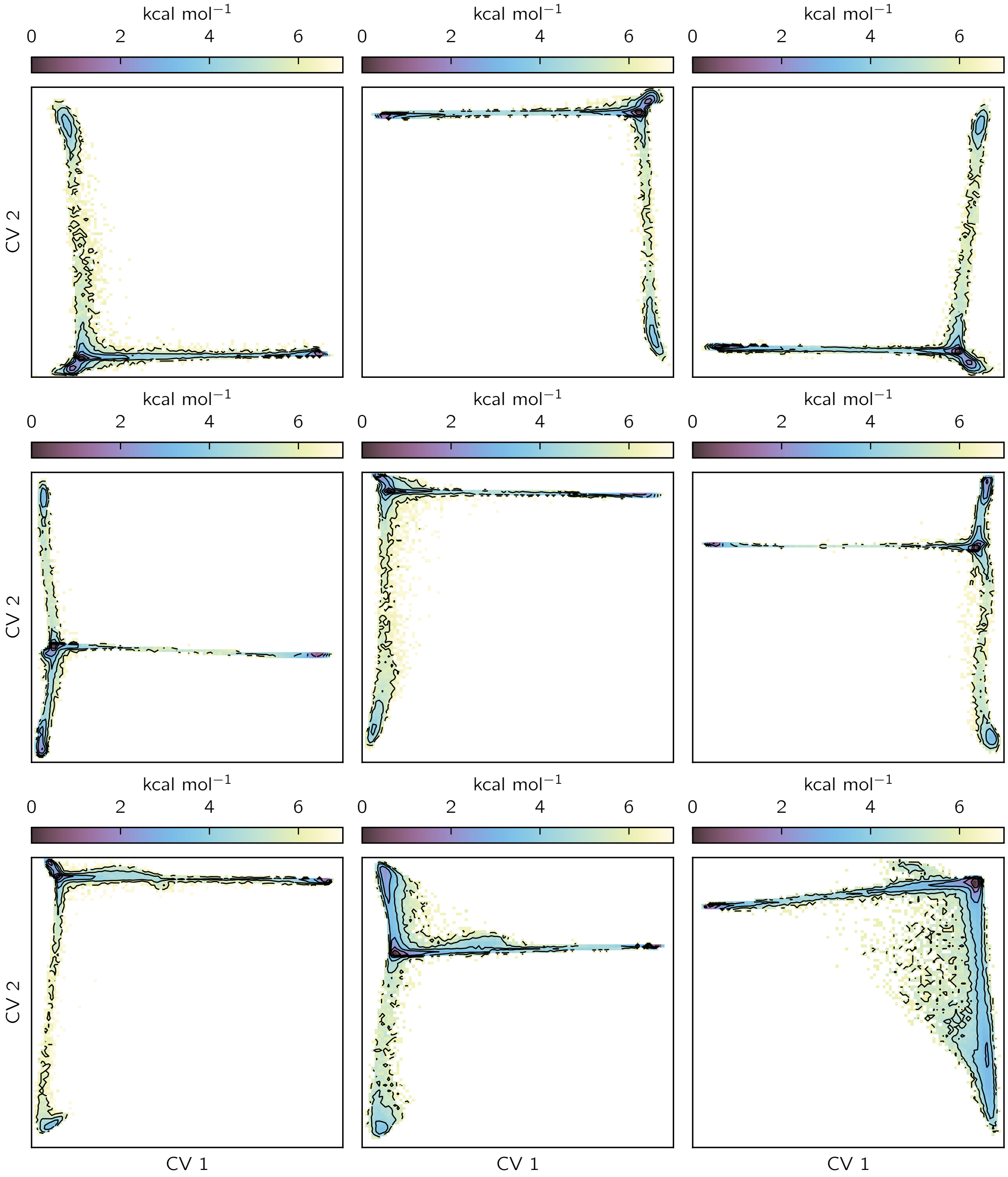


FIG. S4. PMFs as a function of VAMP CVs for trp-cage. From top to bottom, VAMPnets were trained with no token mixer (Sum), SubMixer, or SubFormer. Each column shows the result from a single training run. Contours are drawn every 1 kcal/mol.

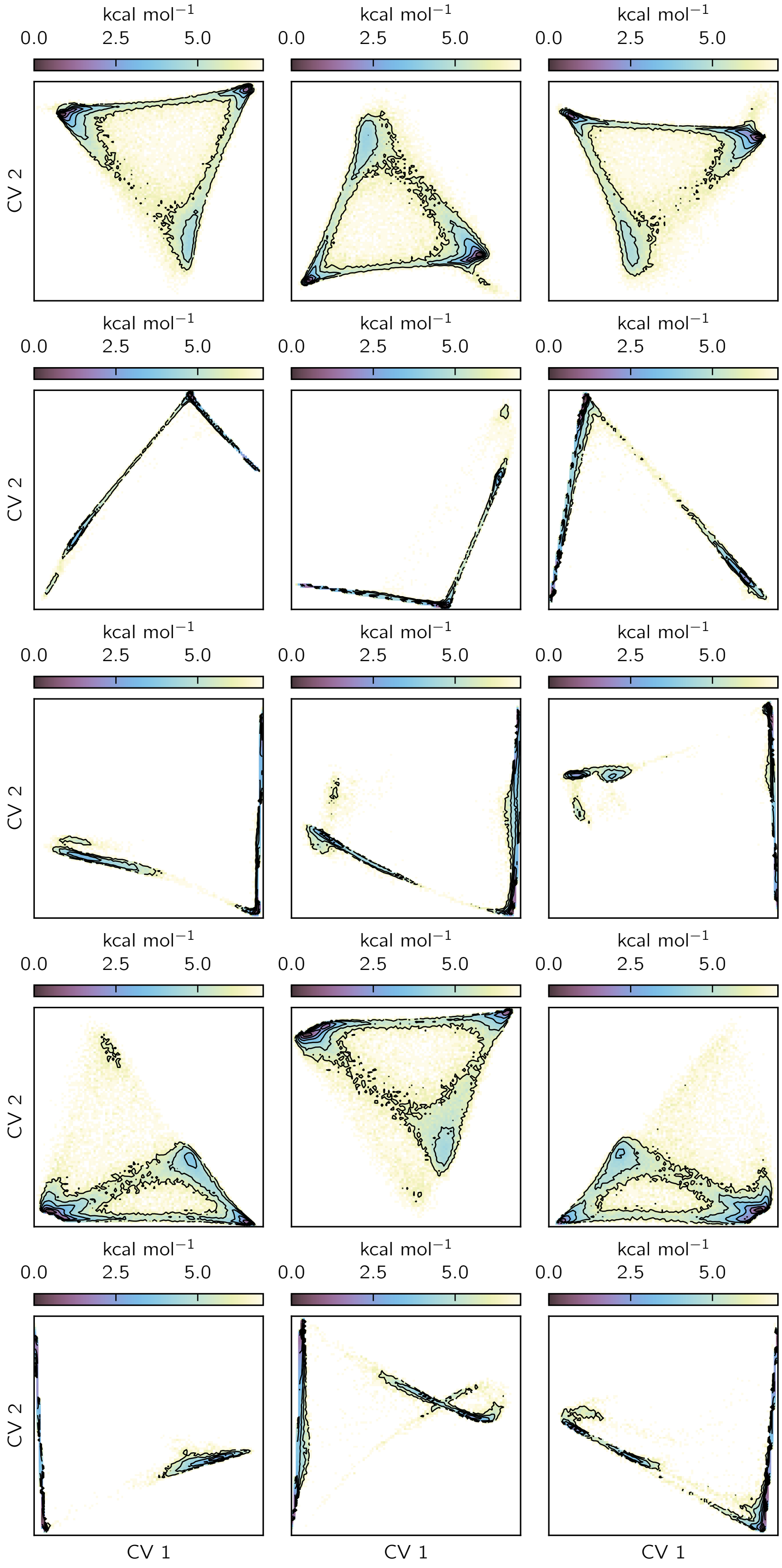


FIG. S5. PMFs as a function of VAMP CVs for villin. From top to bottom, VAMPnets were trained with no token mixer (Sum), SubMixer, SubMixer-GVP, SubFormer, or SubFormer-GVP. Each column shows the result from a single training run. Contours are drawn every 1 kcal/mol.

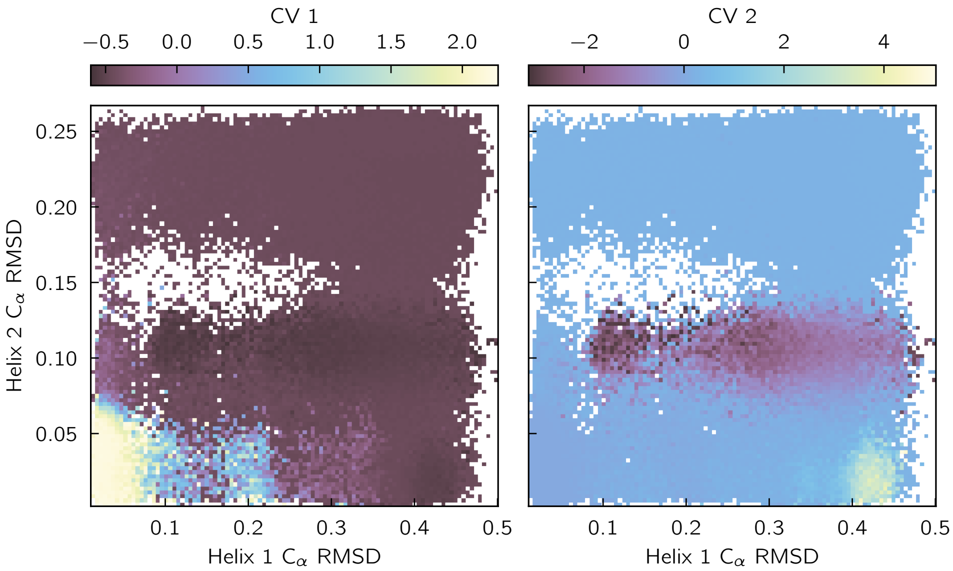


FIG. S6. Trp-cage VAMPnet (with SubMixer) CVs as a function of two physical coordinates: $C_\alpha$ RMSD of helix 1 (residues 2–9) and $C_\alpha$ RMSD of helix 2 (residues 11–14). The $C_\alpha$ RMSDs were computed with respect to the PDB structure 2JOF[64].

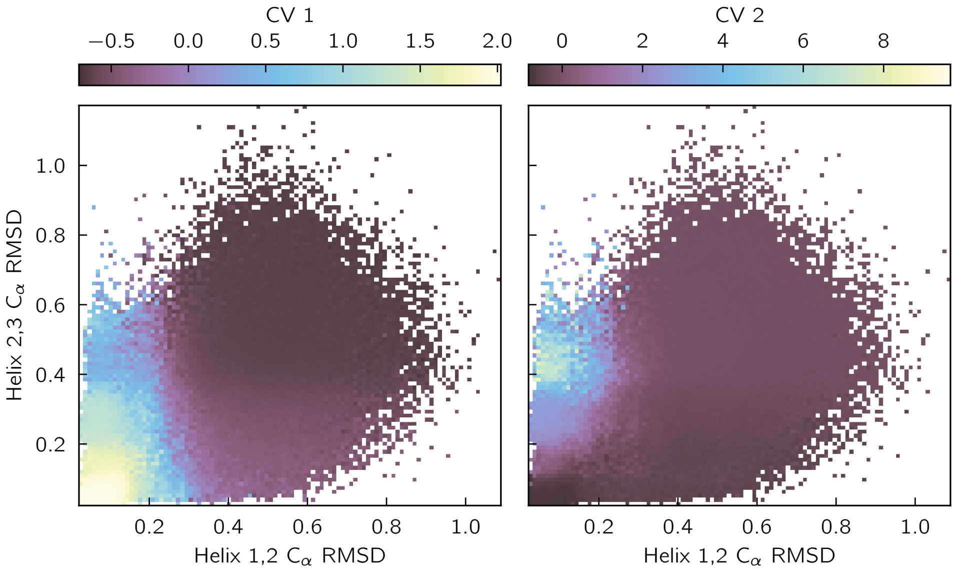


FIG. S7. Villin VAMPnet (with SubFormer) CVs as a function of two physical coordinates: $C_\alpha$ RMSD of helices 1 and 2 (residues 3–10 and 14–19), and $C_\alpha$ RMSD of helices 2 and 3 (residues 14–19 and 22–32). The $C_\alpha$ RMSDs were computed with respect to the PDB structure 2F4K[67].

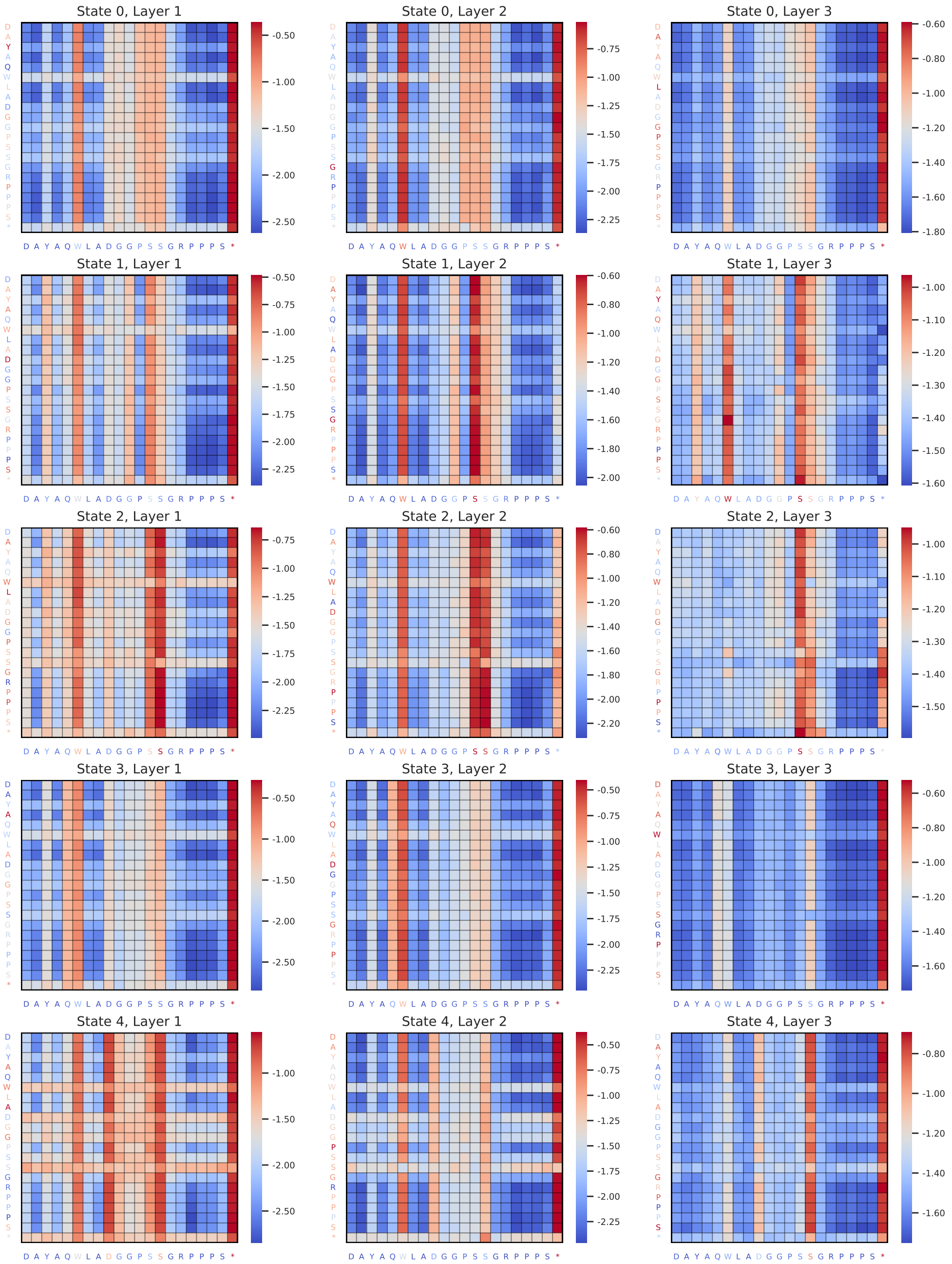


FIG. S8. Log-scaled attention weight heatmaps for trp-cage SPIB states 0 to 4 from three layers of SubFormer-GVP. Each subplot displays attention weights with color-coded tick labels based on normalized sums. Colorbars indicate log-scaled attention values.

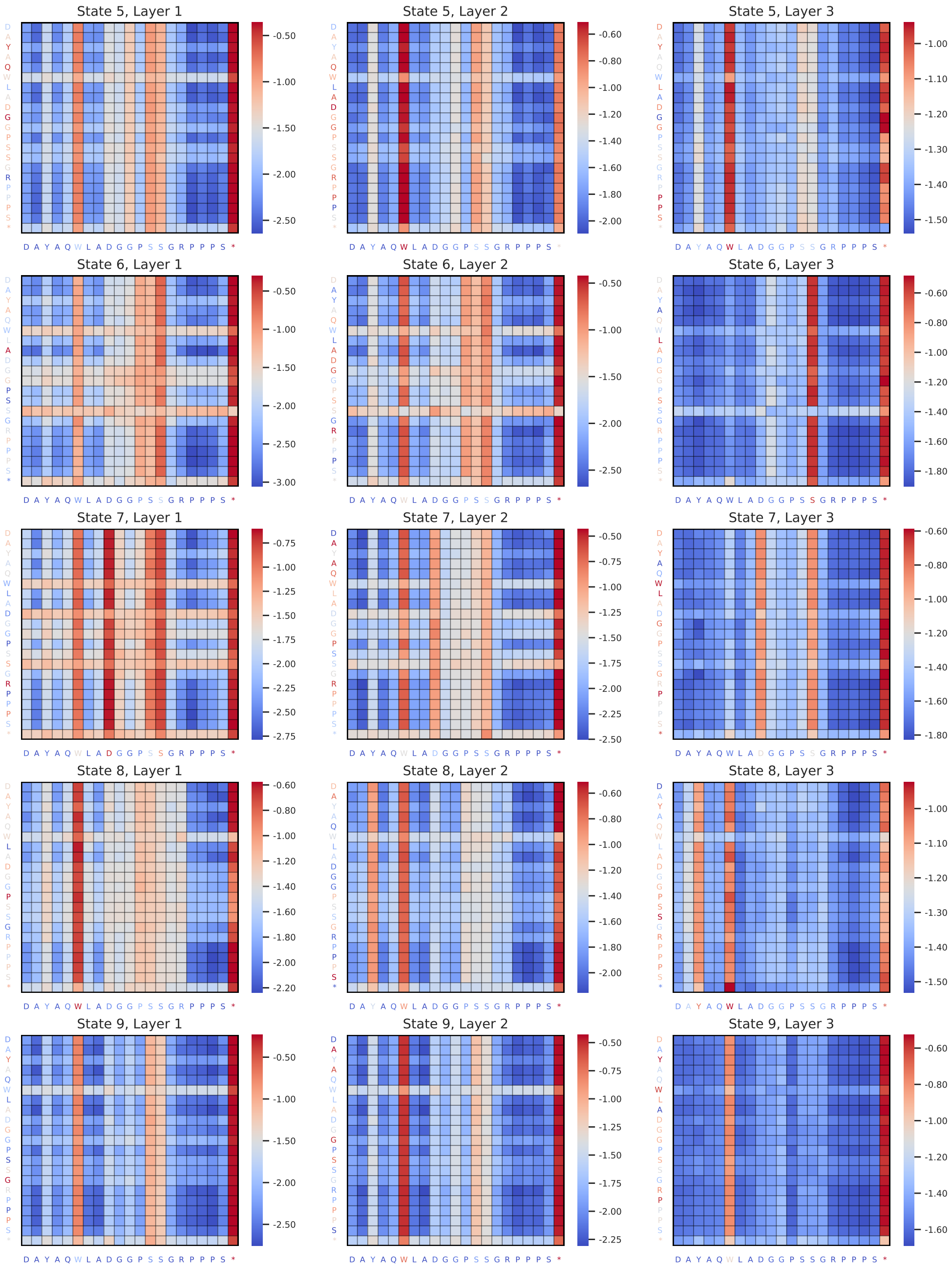

FIG. S9. Log-scaled attention weight heatmaps for trp-cage SPIB states 5 to 9 from three layers of SubFormer-GVP. Each subplot displays attention weights with color-coded tick labels based on normalized sums. Colorbars indicate log-scaled attention values.

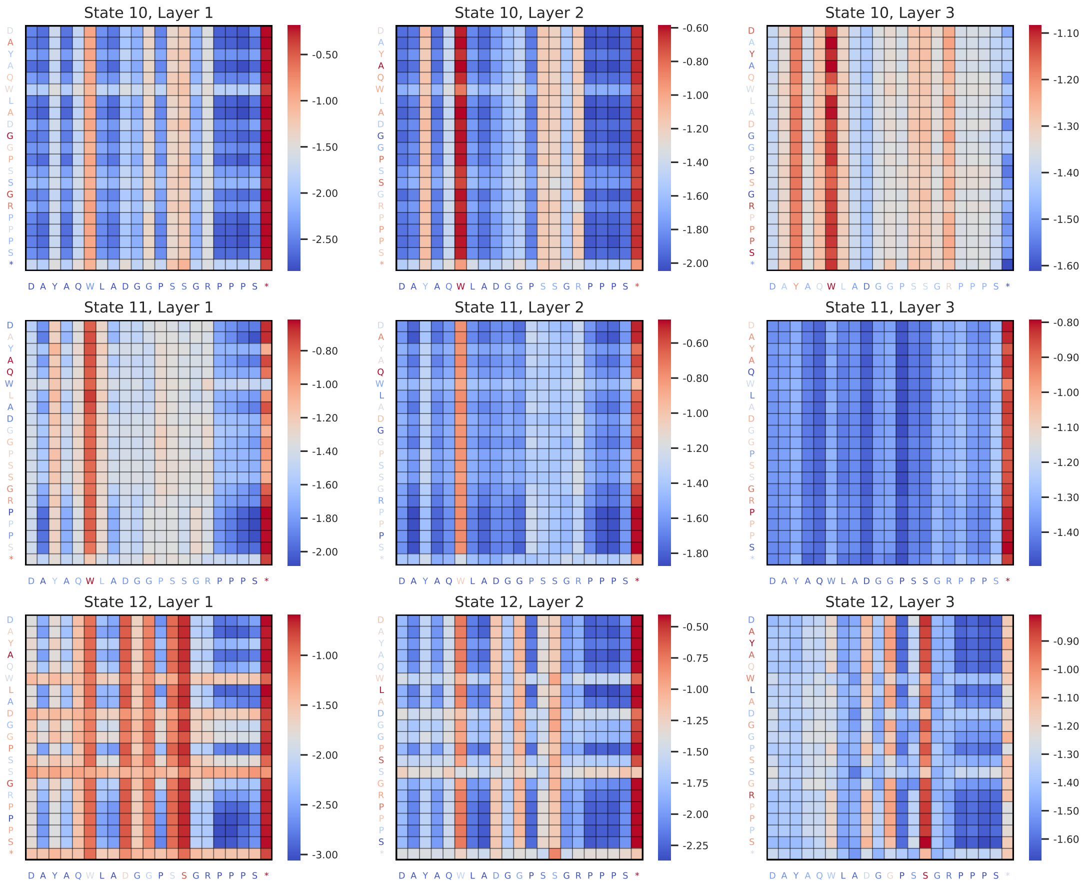


FIG. S10. Log-scaled attention weight heatmaps for trp-cage SPIB states 10 to 12 from three layers of SubFormer-GVP. Each subplot displays attention weights with color-coded tick labels based on normalized sums. Colorbars indicate log-scaled attention values.

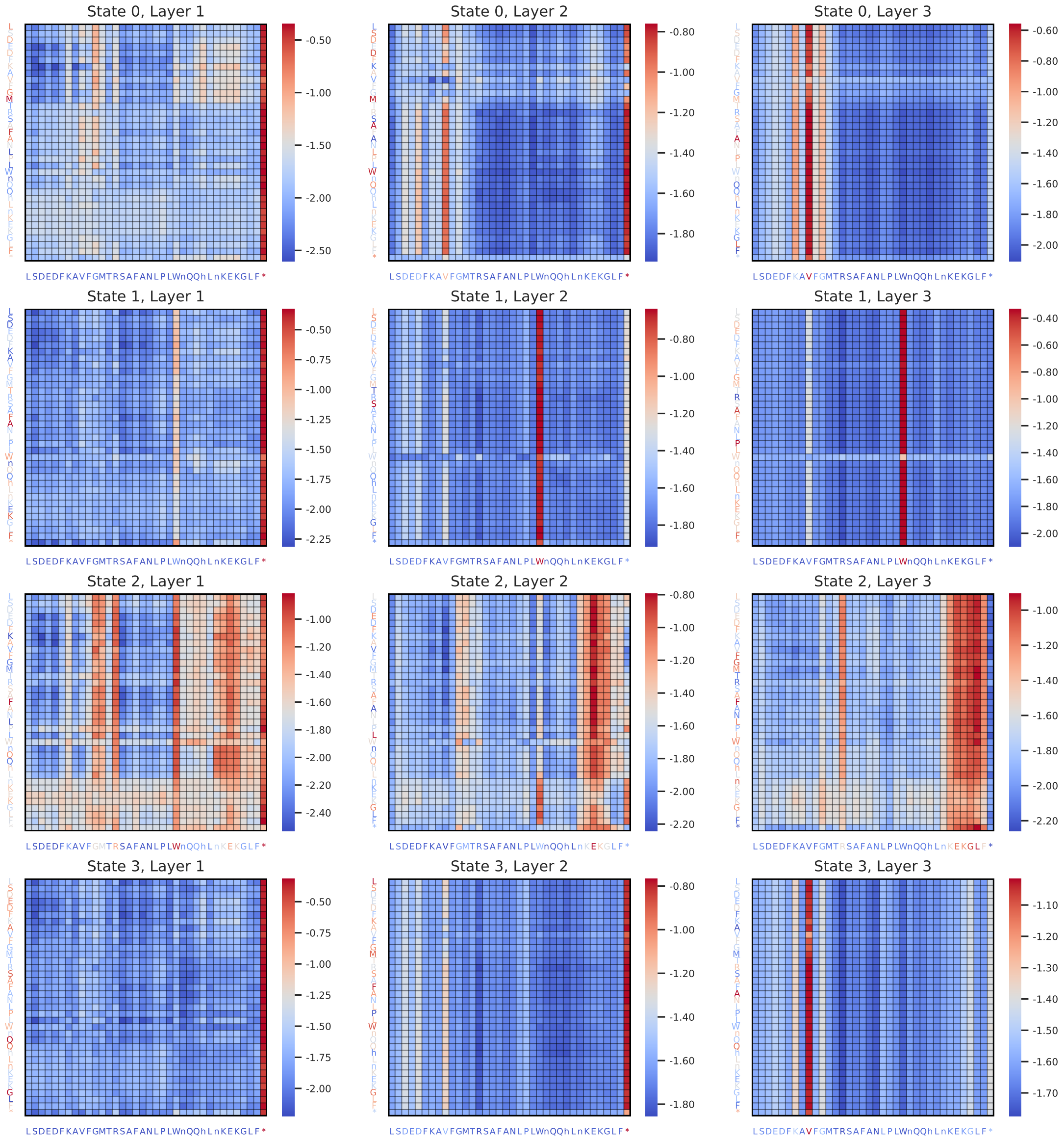


FIG. S11. Log-scaled attention weight heatmaps for villin SPIB states 0 to 3 from three layers of SubFormer-GVP. Each subplot displays attention weights with color-coded tick labels based on normalized sums. Colorbars indicate log-scaled attention values.

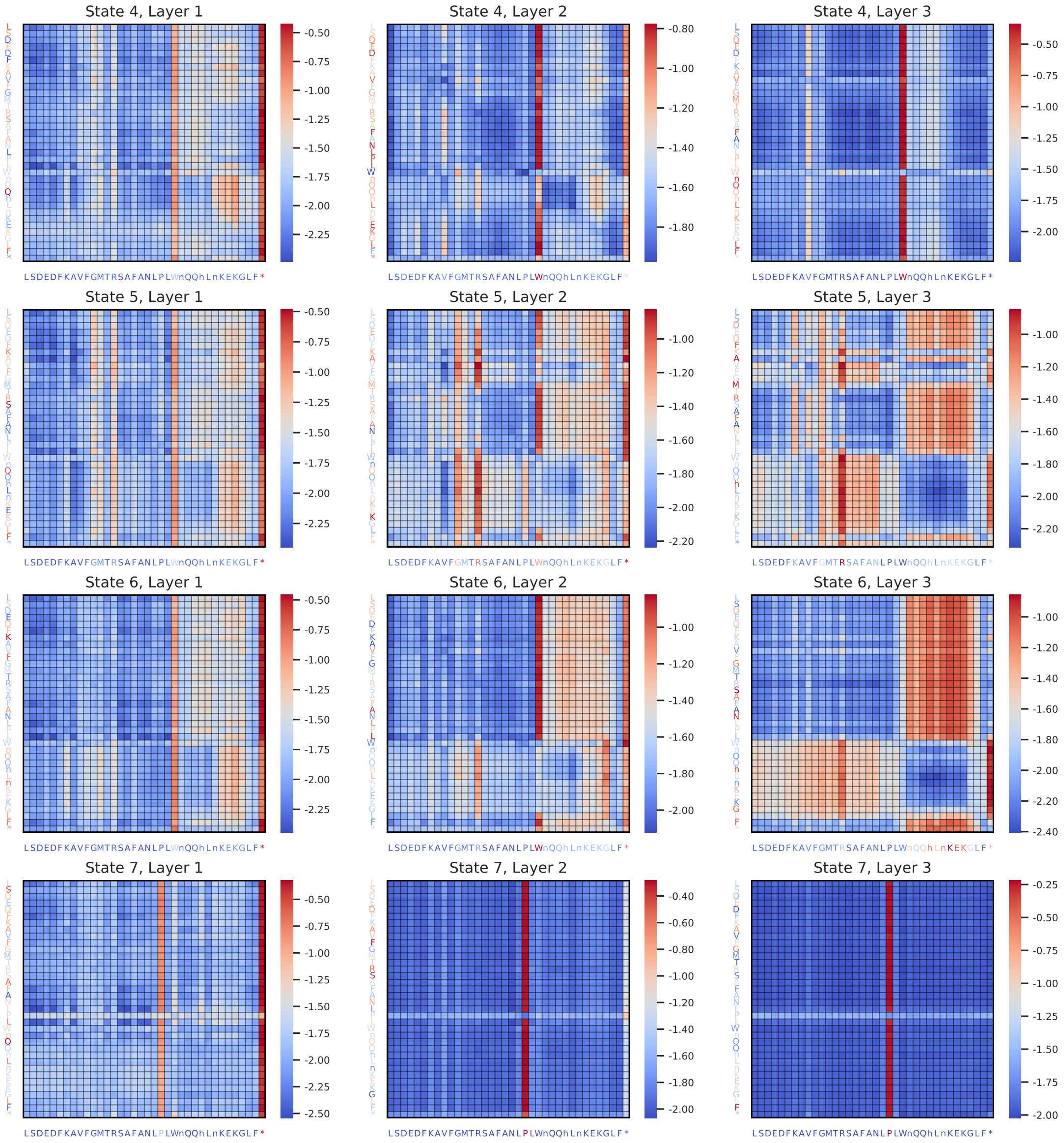


FIG. S12. Log-scaled attention weight heatmaps for villin SPIB states 4 to 7 from three layers of SubFormer-GVP. Each subplot displays attention weights with color-coded tick labels based on normalized sums. Colorbars indicate log-scaled attention values.

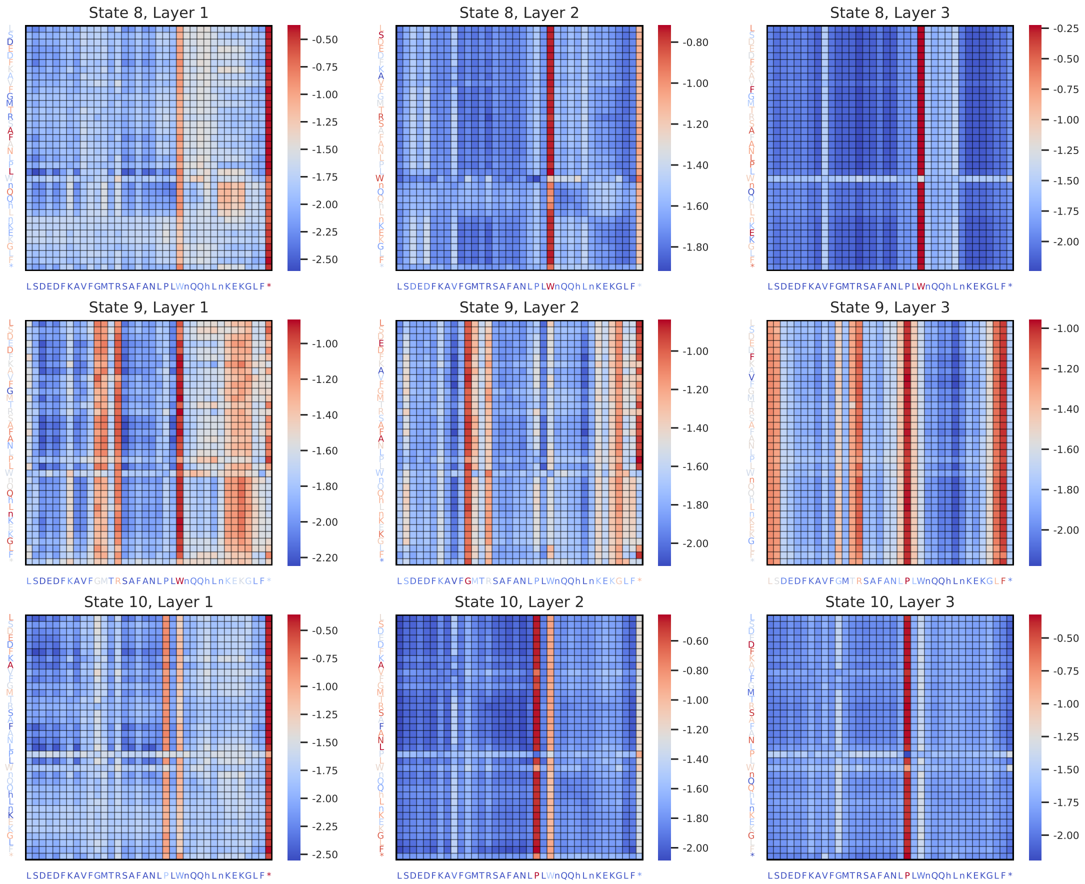


FIG. S13. Log-scaled attention weight heatmaps for villin SPIB states 8 to 10 from three layers of SubFormer-GVP. Each subplot displays attention weights with color-coded tick labels based on normalized sums. Colorbars indicate log-scaled attention values.

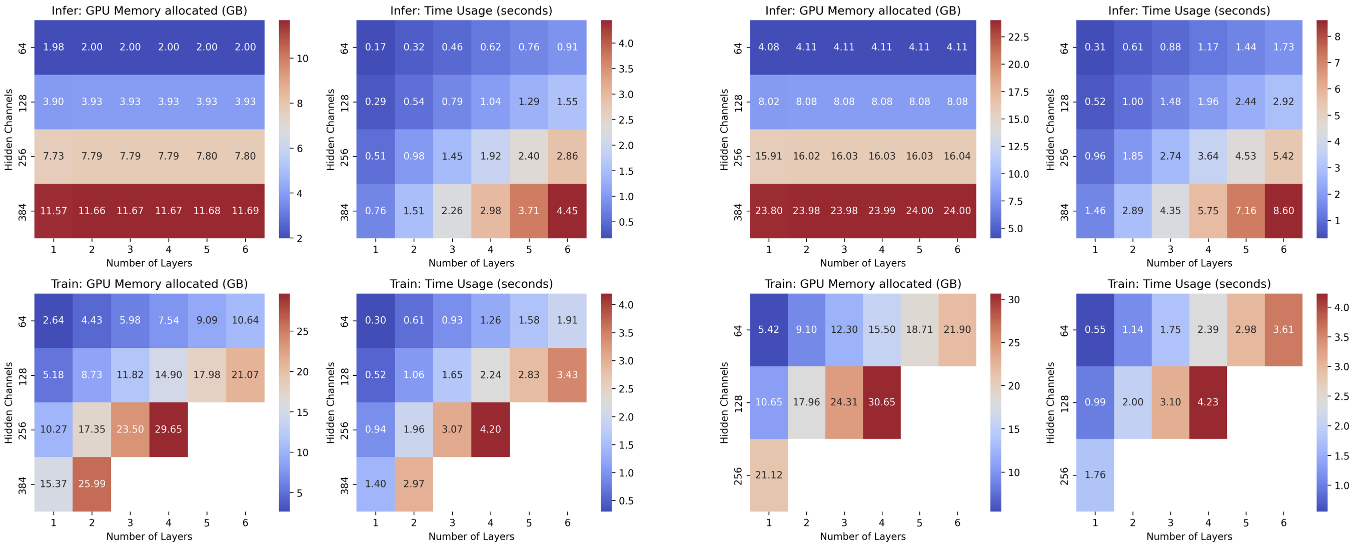


FIG. S14. Comparison of time and GPU memory usage of TorchMD-ET in training and inference modes on the trp-cage (left) and villin (right) subsets, each consisting of 2000 frames with 100 frames per batch. The measurements are done without specific objective functions for usage benchmarking purposes. The model's performance is evaluated across varying numbers of hidden channels (64, 128, 256, 384) and layers (1–6). The heatmaps on the left show GPU memory allocated in gigabytes (GB), while the heatmaps on the right depict time usage in seconds. The time represents one forward pass for inference mode and one forward plus one backward pass for training mode. Measurements were performed on an NVIDIA A100 GPU with 40G of memory. Missing values are due to out-of-memory errors for certain configurations.
